# Treatment Response and Conditional Survival in Advanced Pancreatic Cancer Patients Treated with FOLFIRINOX: A Multicenter Cohort Study

**DOI:** 10.1155/2022/8549487

**Published:** 2022-07-07

**Authors:** Fleur van der Sijde, Jacob L. van Dam, Bas Groot Koerkamp, Brigitte C. M. Haberkorn, Marjolein Y. V. Homs, Daniëlle Mathijssen, Marc G. Besselink, Johanna W. Wilmink, Casper H. J. van Eijck

**Affiliations:** ^1^Department of Surgery, Erasmus MC, University Medical Center Rotterdam, Rotterdam, Netherlands; ^2^Department of Medical Oncology, Maasstad Hospital, Rotterdam, Netherlands; ^3^Department of Medical Oncology, Erasmus MC, University Medical Center Rotterdam, Rotterdam, Netherlands; ^4^Department of Medical Oncology, Franciscus Gasthuis, Rotterdam, Netherlands; ^5^Department of Surgery, Cancer Center Amsterdam, Amsterdam UMC, University of Amsterdam, Amsterdam, Netherlands; ^6^Department of Medical Oncology, Cancer Center Amsterdam, Amsterdam UMC, University of Amsterdam, Amsterdam, Netherlands

## Abstract

**Background:**

FOLFIRINOX chemotherapy is the current Dutch standard of care for locally advanced (LAPC) and metastatic pancreatic cancer (PDAC) patients with good performance status. The objective of this study was to evaluate real-world response rates and survival in advanced PDAC and to assess conditional survival after FOLFIRINOX.

**Methods:**

A multicenter, retrospective cohort study was conducted in four hospitals in the Netherlands. Consecutive patients with LAPC or metastatic PDAC, treated with FOLFIRINOX, were included.

**Results:**

Between 2012 and 2018, 284 patients were included: *n* = 136 with LAPC and *n* = 148 with metastatic PDAC. Objective response rates were similar in both the groups: 14.0% in LAPC and 18.2% in metastatic patients. The disease control rate was higher in LAPC patients (77.2%) compared to metastatic PDAC (51.4%, *P* < 0.001). Median overall survival (OS) in LAPC patients was 12.7 months (95% CI 11.4–14.1 months). Their 2-year survival probability increased from 14% to 26% one year after the completion of FOLFIRINOX. Median OS in metastatic PDAC patients was 8.1 months (95% CI 6.5–9.6 months); 2-year survival probability increased from 10% to 29% after one year. *Discussion*. Our study provides real-world estimates of response rates, survival, and conditional survival in patients with advanced PDAC treated with FOLFIRINOX. These results are useful for patient counseling and clinical decision making.

## 1. Introduction

Pancreatic ductal adenocarcinoma (PDAC) is a cancer known for its poor prognosis. Eighty percent of patients present with advanced disease at the time of diagnosis, meaning they present with locally advanced disease or distant metastases [1]. These patients are not eligible for surgical resection of the tumor, and therefore, systemic chemotherapy is one of few treatment options. FOLFIRINOX chemotherapy (a combination of fluorouracil, leucovorin, irinotecan, and oxaliplatin) is the current standard of care in the Netherlands for patients with a good performance status (World Health Organization grade 0-1). Although FOLFIRINOX has been associated with improved survival compared to other regimens [2, 3], response rates still are disappointing, [4] and patients and physicians are sometimes reluctant to start FOLFIRINOX because of the high toxicity rate [2, 5]. However, reliable or useful data on response rates and survival benefit are lacking; most studies in the literature report data on metastatic patients only, or report outcomes for all disease stages combined, which are difficult to interpret for patient counseling. Moreover, patient outcomes reported from (randomized) clinical trials might be biased by patient selection before entry into the study. Therefore, real-world data are necessary to properly inform patients about expected chemotherapy outcomes and enable shared decision making. Similarly, it is important to be able to inform patients about their future perspectives, also after completion of treatment. Survival probabilities might shift considerably during follow-up, for example, after completion of FOLFIRINOX.

Conditional survival (CS) is the survival probability of patients who have already survived a certain period. CS is most relevant in the assessment of patients with cancers associated with high mortality rates early after diagnosis, such as PDAC. Prognosis is disproportionately negatively influenced by early deaths [6, 7]. Sharing personalized survival probability estimates are important to share with patients and may impact their future decision making, quality of life, and mental wellbeing [6].

The objective of this study was to evaluate the real-world outcomes of FOLFIRINOX response and survival in advanced PDAC patients and to assess CS, using the data from a multicenter, retrospective cohort study.

## 2. Methods

### 2.1. Study Design

This multicenter, retrospective study was conducted at four hospitals in the Netherlands: Erasmus University Medical Center (Rotterdam), Amsterdam University Medical Center (Amsterdam), Maasstad Hospital (Rotterdam), and Franciscus Gasthuis (Rotterdam/Schiedam).

### 2.2. Patient Selection

By searching the hospital pharmacy records, we identified all consecutive patients who had received FOLFIRINOX chemotherapy for LAPC or metastatic PDAC between January 2012 and December 2018. Pancreatic malignancy was histologically confirmed in all patients. Locally advanced PDAC was defined according to the Dutch Pancreatic Cancer Group criteria; LAPC was defined as >90 degrees of arterial contact or >270 degrees of venous contact [8]. Patient characteristics such as age, sex, stage of disease, FOLFIRINOX chemotherapy specifics, laboratory results, CT scan evaluations, and follow-up data were retrieved from medical records. The study protocol was approved by the medical ethics review boards of all participating hospitals.

### 2.3. FOLFIRINOX Treatment

Patients received FOLFIRINOX chemotherapy every two weeks in the following dosages: oxaliplatin 85 mg/m^2^ infused over 120 min, immediately followed by leucovorin at a dose of 400 mg/m^2^ infused over 120 min with the addition, after 30 min, of irinotecan 180 mg/m^2^ infused over 90 min, followed by fluorouracil 400 mg/m^2^ intravenous bolus, followed by 2400 mg/m^2^ continuous infusion for 46 hours. FOLFIRINOX chemotherapy was discontinued if progression of disease was evident, at the patient's request, or in case of unacceptable toxicity.

Locally advanced patients were scheduled for eight to twelve cycles of FOLFIRINOX chemotherapy. Patients showing complete or partial response or stable disease after chemotherapy might receive stereotactic radiation therapy and/or surgical resection. Some patients participated in the PELICAN trial (Dutch trial register NL4997), in which the stage of the disease is re-evaluated after four cycles of FOLFIRINOX. In this trial, if there is no disease progression after four cycles of FOLFIRINOX, patients are randomized between radiofrequency ablation followed by the continuation of chemotherapy, or chemotherapy only. In both arms, a total of 12 cycles of FOLFIRINOX is given. The standard of care in the Netherlands for metastatic disease patients is twelve cycles of FOLFIRINOX chemotherapy.

In three out of four investigated centers (Erasmus University Medical Center, Maasstad Hospital, and Franciscus Gasthuis), every patient received granulocyte colony-stimulating factor (G-CSF) to prevent chemotherapy-induced neutropenia starting at the first cycle of FOLFIRINOX. G-CSF was given in a single bolus of 6 mg of lipegfilgastrim or pegfilgrastim, 24–48 hours after the start of each chemotherapy cycle. In the Amsterdam University Medical Center, only patients that developed grade 3–4 neutropenia during FOLFIRINOX would receive G-CSF upon the next cycle of FOLFIRINOX. There is no national consensus on the use of G-CSF during FOLFIRINOX.

### 2.4. Treatment Response Evaluation

A CT scan to evaluate the tumor response to treatment was performed after every fourth cycle of FOLFIRINOX or earlier, if patients showed clinical signs of tumor progression or treatable disease symptoms, such as biliary obstruction, that could be diagnosed with CT scans. The diameters of target lesions were determined by experienced radiologists and treatment response was reported according to the Response Evaluation Criteria in Solid Tumours (RECIST) 1.1 criteria. The final response was defined as the treatment response measured on the last evaluation CT scan. The time point of this last CT scan varied due to differences between patients in the number of chemotherapy cycles received. The objective response rate (ORR) was defined as the proportion of patients with complete response or partial response to treatment. The disease control rate (DCR) was defined as the proportion of patients with complete response, partial response, or stable disease.

### 2.5. Statistical Analysis

Categorical patient characteristics (e.g., sex, location of the tumor, and RECIST response outcomes) were compared between LAPC and metastatic disease patients with Pearson's Chi-squared tests. Continuous patient characteristics (e.g., age, baseline CA 19-9, and number of FOLFIRINOX cycles) were not normally distributed and were therefore compared with the Mann–Whitney *U* tests.

Overall survival (OS) was calculated as the time between the start of chemotherapy and cancer-related death, progression-free survival (PFS) as the time between start of chemotherapy, and radiologic or histologic confirmation of disease progression. Differences in survival between LAPC and metastatic disease patients and between the different RECIST response outcome patient groups were estimated with Kaplan–Meier curves and compared with log-rank tests.

Conditional survival (CS) was defined as the probability that a patient would survive an additional number of months or years after already having survived a certain time. CS was calculated using Kaplan–Meier survival estimates with the formula: CS_(x/y)_ = S_(x/y)/_S_(x)_, with *S* = survival estimate, *x* = number of months survived and *y* = number of additional months. For example, to estimate the CS for surviving one additional year for patients who already had survived one year, CS_(12/12)_ is calculated by dividing the 2-year Kaplan–Meier survival estimate S_(24)_ by the 1-year Kaplan–Meier survival estimate S_(12)_ [7, 9, 10]

All tests were performed two-sided, and *P* values <0.05 were considered statistically significant. Data were analyzed using SPSS (version 25.0; IBM, Armonk, NY, USA) and R software, version 4.04.

## 3. Results

### 3.1. Patient Characteristics

The data of 284 consecutive patients–136 diagnosed with LAPC and 148 with metastatic PDAC–who started FOLFIRINOX were included in the analyses.

**Figure 1:**
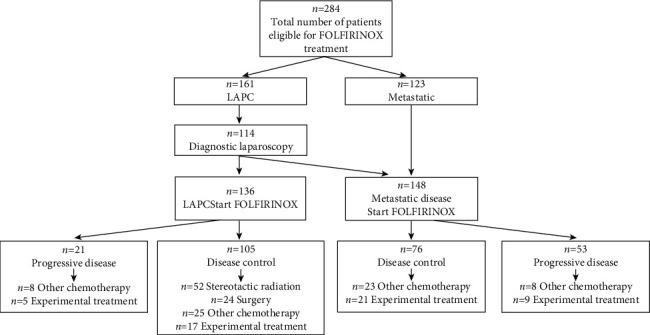
Flowchart presenting the patients with locally advanced (LAPC, *n* = 161) and metastatic pancreatic cancer (*n* = 123) eligible for FOLFIRINOX, the treatment response outcome based on the RECIST criteria (*n* = 21 progressive LAPC, *n* = 105 disease control LAPC, and *n* = 53 progressive metastatic, *n* = 76 disease control metastatic patients), and additional treatment received after first-line chemotherapy, e.g., radiation, surgery, other chemotherapy, or experimental (immune) treatment. Patients could have received more than one type of additional treatment after FOLFIRINOX.

Patient characteristics are reported in Table 1. The median serum CA 19-9 levels prior to start of FOLFIRINOX in patients with metastatic PDAC was 919 kU/L (IQR 125–5622 kU/L) and in LAPC patients was 314 kU/L (IQR 92–1131 kU/L, *P* < 0.001). In patients with metastatic PDAC, the primary tumor was more often located in the tail of the pancreas (35 patients (23.6%) vs 8 patients (5.9%), *P* < 0.001). In the metastatic patient group, 122/148 patients (82.4%) showed metastases at PDAC diagnosis; the other 26 patients (17.6%) received FOLFIRINOX chemotherapy upon metastasis after surgical resection of the primary tumor. Of those patients, 15 showed local recurrence at the surgical resection site without distant metastases. Twenty-five patients, initially identified as having LAPC, were found to have metastatic disease on diagnostic laparoscopy. The median number of FOLFIRINOX cycles received did not significantly differ between the LAPC and metastatic PDAC groups. The majority of LAPC patients (63.2%) received additional treatment (e.g. stereotactic radiation, surgery, other chemotherapy, immune therapy) after FOLFIRINOX chemotherapy, whereas only 34.5% of metastatic patients received additional treatment (*P* < 0.001). In LAPC, 33/136 (24.3%) patients underwent any second-line chemotherapy; in metastatic disease patients, this was 31/148 (21.0%). In this cohort, the resection rate in LAPC patients was 24/136 (17.8%). Eight of the resected patients received adjuvant gemcitabine chemotherapy. None of the patients with metastatic disease underwent surgery. A flowchart of LAPC and metastatic disease patients eligible for FOLFIRINOX and the additional therapies received after FOLFIRINOX is presented in Figure 1.

### 3.2. Comparison of Chemotherapy Response Rates

FOLFIRINOX response at the different evaluation time points and final ORR and DCR are presented in Table 2. In total, 28 patients (LAPC, *n* = 10, 7.4%; metastatic disease, *n* = 18, 12.2%) stopped treatment due to unacceptable toxicity of FOLFIRINOX before CT evaluation was performed. At the first CT evaluation, 35/148 (23.6%) of metastatic patients and 14/136 (10.3%) of LAPC patients showed progressive disease (*P*=0.002), as determined by RECIST 1.1 criteria. In 41/136 (30.2%) of LAPC and 51/148 (34.5%) of metastatic patients, no data from the second CT evaluation were available, mostly due to preliminary termination of chemotherapy treatment upon patients' request. After twelve cycles of FOLFIRINOX, metastatic patients showed again more often progressive disease (8.1% vs 0%, *P* < 0.001).

In most patients in the LAPC group, the final response outcome was stable disease (63.2%, 86/136), and in an additional 14% (19/136) of patients, the final response outcome was partial response. In LAPC, 15.4% (21/136) of patients showed progressive disease during or immediately after FOLFIRINOX chemotherapy. In metastatic disease patients, 33.1% (49/148) showed stable disease, 18.2% (27/148) partial response, and 35.8% (53/148) progressive disease (*P* < 0.001). None of the patients showed a complete response. The ORR was similar between LAPC (14.0%) and metastatic disease patients (18.2%, *P* = 0.329), but the DCR was significantly higher in LAPC patients (77.2% vs 51.4%, *P* < 0.001).

Biochemical response to treatment could, unfortunately, not be determined, since tumor marker analyses during and after chemotherapy are not included in standard clinical practice in the Netherlands.

### 3.3. The Impact of Disease Stage and Treatment Response on Survival

**Figure 2:**
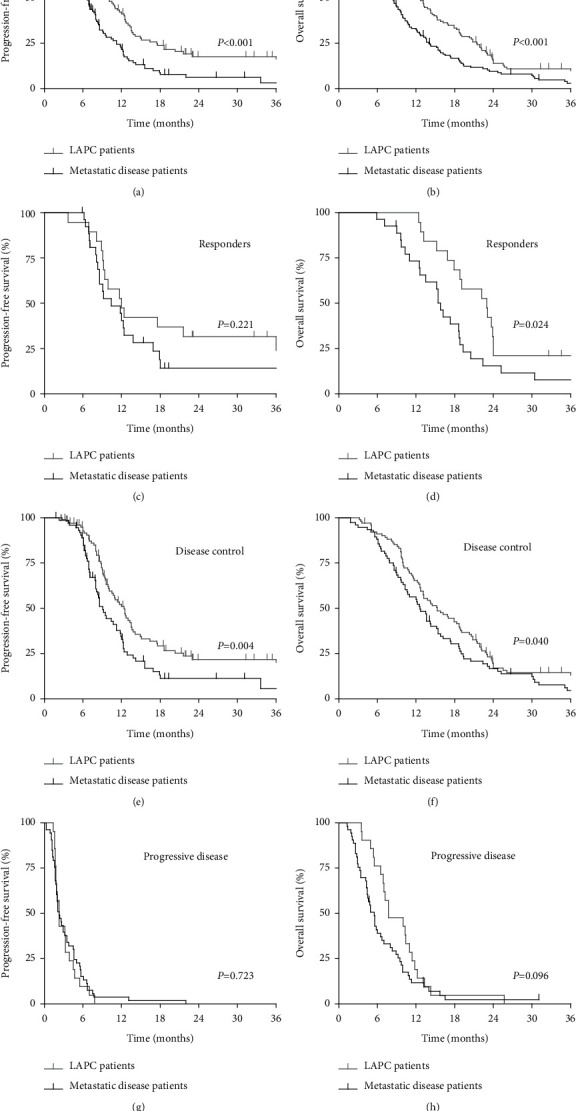
Kaplan–Meier estimates for progression-free survival and overall survival of patients with locally advanced pancreatic cancer (LAPC) and metastatic pancreatic cancer after treatment with FOLFIRINOX. (a, b) Total cohorts of LAPC (*n* = 136) and metastatic disease patients (*n* = 148). (c, d) LAPC (*n* = 19) versus metastatic disease patients (*n* = 27) with the partial response after FOLFIRINOX treatment, according to the RECIST 1.1 criteria (e, f) LAPC (*n* = 105) versus metastatic disease patients (*n* = 76) with disease control after FOLFIRINOX, including partial response or stable disease. (g, h) LAPC (*n* = 21) versus metastatic disease patients (*n* = 53) with progressive disease during or immediately after FOLFIRINOX.

The median OS was 12.7 months (95% CI 11.4–14.1 months) in LAPC patients and 8.1 months (95% CI 6.5–9.6) in patients with metastatic PDAC (*P* < 0.001). Kaplan–Meier curves are shown in Figure 2. The median PFS was longer in LAPC patients (10.0 months, 95% CI 8.5–11.5 months) versus metastatic disease patients (6.7 months, 95% CI 6.0–7.4 months, *P* < 0.001). LAPC patients with partial response showed significantly longer median OS (23.0 months, 95% CI 17.3–28.7 months vs 15.8 months, 95% CI 14.6–17.0 months, *P* = 0.024), though not significantly longer median PFS (12.0 months, 95% CI 8.5–15.5 months vs 10.4 months, 95% CI 6.0–14.8 months, *P* = 0.221) compared to metastatic patients with a partial response to FOLFIRINOX treatment. When comparing disease control patients, the LAPC patient group again was associated with better median OS (15.3 months, 95% CI 12.0–18.7 months vs 12.6 months, 95% CI 10.4–14.8 months, *P* = 0.040) and median PFS (12.4 months, 95% CI 10.7–14.1 months vs 9.1 months, 95% CI 7.8–10.4 months, *P* = 0.004) compared to metastatic disease patients. However, for patients with progressive disease during FOLFIRINOX chemotherapy, median OS (7.7 months, 95% CI 3.5–11.8 months vs 5.5 months, 95% CI 4.5–6.6 months, *P* = 0.096) did not significantly differ between LAPC and metastatic disease patients.

### 3.4. Conditional Survival Analysis

**Figure 3:**
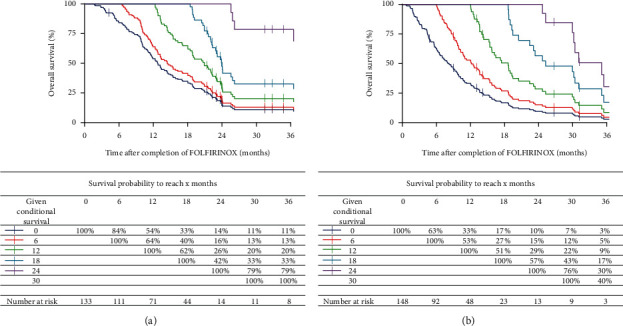
Kaplan–Meier estimates for conditional survival up to three years, given a survival of 6–30 months after completion of FOLFIRINOX in locally advanced pancreatic cancer (LAPC) patients (a) and metastatic disease patients (b).

Conditional survival curves are presented in Figure 3. In LAPC patients, the probability of surviving two years increased from 14% at the start of FOLFIRINOX to 16%, 26%, and 42% with every additional six months survived up to 1.5 years. In metastatic patients, the probability of achieving 2-year survival increased from 10% to 15%, 29%, and 57%. The probability to survive one additional year, or 1-year CS, was 54% in LAPC patients at the start of FOLFIRINOX, then decreased to 26% after one year and increased to 79% after two years. In metastatic patients, the 1-year CS was 33%, then decreased to 29% after one year, and was 30% after two years.

## 4. Discussion

In this retrospective cohort study, we evaluated differences in FOLFIRINOX response outcome and survival after FOLFIRINOX in patients with either LAPC or metastatic PDAC. We found similar ORR in the two patient groups but significantly higher DCR in LAPC patients (77.2%) compared to patients with metastatic PDAC (51.4%). As expected, the median OS and PFS in LAPC patients were better than in patients with metastatic PDAC. With an overall response rate of >50%, physicians and patients might be encouraged to start with FOLFIRINOX treatment, because the majority of the patients (40–70%) with advanced PDAC do not receive any palliative chemotherapy at this moment [11, 12]. Also, other real-world data sets, for example, the Danish Pancreatic Cancer Database, show that especially FOLFIRINOX is associated with much better outcomes in terms of survival as compared to best supportive care [11, 12]. In addition, LAPC patients report good quality of life after eight cycles of FOLFIRINOX, measured with validated questionnaires [13].

This is the first study on CS after chemotherapy in advanced PDAC patients. Previous studies with CS analyses included patients with resectable disease [6, 10, 14], which is the patient group with overall the highest survival rate. Real-world data from advanced PDAC patients undergoing chemotherapy treatment are, unfortunately, often lacking or hard to obtain. Therefore, patients are currently being informed about chemotherapy outcome based on information from clinical trials, performed in highly selected patient cohorts. The benefit of treatment might be overestimated due to exclusion of patients with poor prognosis in clinical trials. The survival of patients included in our unselected cohort is far shorter than the reported median survival in literature. For example, a meta-analysis of 13 clinical trials describing FOLFIRINOX in LAPC patients reported a median OS of more than two years [15], while our results show a median OS of just 12.7 months in patients with similar stage of the disease. From multiple clinical trials, the median OS in metastatic PDAC patients has been estimated at 10-11 months [4]. In this retrospective study, however, we found a median OS of 8 months with FOLFIRINOX. Furthermore, in the Netherlands, second-line chemotherapy or experimental treatment in clinical trials in case of progression are not often offered, which might partly explain these poor results.

Locally advanced stage of PDAC is considered to be different from metastatic disease stage, with different treatment approaches and, more importantly, different expected outcomes. Therefore, we presented response to chemotherapy data and survival data separately. However, our study failed to account for the possible effect of other treatments used in survival, such as other chemotherapy regimens, resection, radiotherapy, or irreversible electroporation (IRE). Furthermore, by using the “Dutch Pancreatic Cancer Group criteria” for PDAC staging and resectability: >90 degrees of arterial contact or >270 degrees of venous contact, we might have included a number of patients that would have been staged as a borderline resectable disease based on the more widely accepted and used staging systems: NCCN and AHPBA/SSO/SSAT criteria.

In our cohort, 17.8% of LAPC patients were resected after FOLFIRINOX, which is at the lower end of the results from published literature. In a patient-level meta-analysis of FOLFIRINOX for LAPC from high-volume centers, the resection rate was 25.9% [15] and the median resection rate after completion of neoadjuvant treatment was 25% in a recently published systematic review by Attard et al. [16]. Our lower resection rate could probably be explained because arterial resections were not considered to be performed in the Netherlands until recently.

In addition to OS, we consider CS an important outcome to be discussed with patients scheduled for FOLFIRINOX. Conditional survival analyses in this cohort showed that in both patient groups, the survival probability increased after completion of FOLFIRINOX. Reassessment of survival probability after FOLFIRINOX might benefit patients; informing them about life expectancy can help patients in decision making on future treatment or supportive care.

This study has some limitations. First, the retrospective design of the study might have a biased outcome. Chemotherapy schedules are standardized, though treating physicians might deviate from this at their own discretion, for example, when patients are responding very well to treatment. In this cohort, some patients received more than eight or twelve cycles of FOLFIRINOX for LAPC or metastatic disease, respectively. In addition, patients could have stopped treatment at any time for any reason, not necessarily due to the progression of the disease only. Second, the Dutch standard for FOLFIRINOX treatment is eight cycles for LAPC patients and twelve cycles for patients with metastatic disease. However, this is not the worldwide standard since some centers administer, for example, only four cycles of chemotherapy, while others continue treatment upon the progression of the disease [15]. We presented response rates after four and eight cycles of FOLFIRINOX which can be used for shared decision-making for first-line chemotherapy and additional treatment. However, survival data might not be applicable to patients that received more or less FOLFIRINOX cycles than the patients described in this study. Third, three out of four hospitals participating in this study, of which two academic hospitals, were relatively high-volume centers defined as treating ≥100 patients per 5 years. A previous study has shown that high hospital volume is associated with improved survival of PDAC patients treated with systemic therapy [17]. Fourth, CS is probably also influenced by other factors besides survival time. Increasing survival rates given CS indicate that patients with poor prognostic factors have already died early after FOLFIRINOX and only patients with beneficial tumor characteristics remain. In contrast to studies with CS analyses in patients with resectable PDAC, we do not have any information on tumor characteristics that might be associated with aggressive biological behavior, [10] such as tumor differentiation, invasiveness, and positive lymph nodes. Fifth, the sample sizes, and especially the low number of patients at risk in CS analyses, resulted in wide confidence intervals, particularly for outcomes longer after FOLFIRINOX. Furthermore, in future analysis, the presence of prognostic factors such as clinical response, immune-nutritional parameters, implementation of second-line chemotherapy, surgical resection, and so on, might be more important for clinical decision-making and should be considered as well. Accumulation of survival probability in the future should include such prognostic factors. However, large numbers of recruited patients will be required together with the correct and complete clinical data and patient outcomes. In conclusion, this study describes outcomes of response rate, survival, and conditional survival of patients with locally advanced and metastatic pancreatic cancer treated with FOLFIRINOX. The disease control rate was higher and survival longer in LAPC patients compared to patients with metastatic disease. Overall survival was shorter in this real-world cohort compared to outcomes reported from clinical trials. One year after the start of chemotherapy, the probability to survive one more year is 15% to 30% in patients with advanced PDAC. These clinical results are useful to optimally counsel patients and could help in shared decision making before the start of chemotherapy and during follow-up.

## Figures and Tables

**Table 1:** Baseline and treatment characteristics of patients with locally advanced (LAPC) and metastatic pancreatic cancer.

	All patients *n* = 284 (%)	LAPC *n* = 136 (%)	Metastatic disease *n* = 148 (%)	*P* value
Age, median (range)	62 (31–81)	63 (37–80)	61 (31–81)	0.507
Age > 75 years	51 (18.0)	27 (19.9)	24 (16.2)	0.375
Sex, male	151 (53.2)	69 (50.7)	82 (55.4)	0.431
Baseline CA 19-9 level (kU/L), median (IQR)	457 (105–2044) (*n* = 220)	314 (92–1131) (*n* = 105)	919 (125–5622) (*n* = 115)	<0.001
Location primary tumor				
Head	173 (60.9)	94 (69.1)	79 (53.4)	<0.001
Body	67 (23.6)	33 (24.3)	34 (23.0)	
Tail	43 (15.1)	8 (5.9)	35 (23.6)	
Multifocal	1 (0.4)	1 (0.7)	0 (0)	
Recurrent disease after surgery	26 (9.2)	NA	26 (17.6)	NA
Local recurrent disease after surgery	15 (5.3)	NA	15 (10.1)	NA
Diagnostic laparoscopy prior to the start of FOLFIRINOX	114 (40.1)	89 (65.4)	25 (16.9)	<0.001
Number of FOLFIRINOX cycles, median (IQR)	8 (4–8)	8 (4–8)	7 (3–10)	0.639
Additional therapy after FOLFIRINOX	148 (52.1)	86 (63.2)	51 (34.5)	<0.001
Surgical resection after FOLFIRINOX	24 (8.5)	24 (17.8)	0 (0)	NA
Adjuvant chemotherapy after surgical resection	8 (2.8)	8 (33.3)	NA	NA

CA 19-9 = carbohydrate antigen 19-9; NA = not applicable.

**Table 2:** FOLFIRINOX response outcomes in patients with locally advanced (LAPC) and metastatic pancreatic cancer, based on the RECIST 1.1 criteria.

	LAPC *n* = 136 (%)	Metastatic disease *n* = 148 (%)	*P* value
CT evaluation 1†			
CR	0 (0)	0 (0)	0.002
PR	11 (8.1)	16 (10.8)	
SD	101 (74.3)	79 (53.4)	
PD	14 (10.3)	35 (23.6)	
Unknown	10 (7.4)	18 (12.2)	

CT evaluation 2†			
CR	0 (0)	0 (0)	0.061
PR	18 (13.2)	29 (19.6)	
SD	60 (44.1)	43 (29.1)	
PD	7 (5.1)	8 (5.4)	
Unknown	41 (30.2)	51 (34.5)	
NA	10 (7.4)	17 (11.5)	

Final response outcome‡			
CR	0 (0)	0 (0)	<0.001
PR	19 (14.0)	27 (18.2)	
SD	86 (63.2)	49 (33.1)	
PD	21 (15.4)	53 (35.8)	
Unknown	10 (7.4)	19 (12.8)	

ORR	19/136 (14.0)	27/148 (18.2)	0.329

DCR	105/136 (77.2)	76/148 (51.4)	<0.001

CR = complete response, DCR = disease control rate, NA = not applicable, ORR = objective response rate, PD = progressive disease, PR = partial response, RECIST = response evaluation criteria in solid tumors, and SD = stable disease. †CT scan evaluations were performed after every fourth cycle of FOLFIRINOX, or earlier in case patients showed clinical signs of tumor progression or treatable disease symptoms that could be diagnosed with radiology. ‡Final response outcome was defined as the RECIST 1.1 treatment response measured on the latest available evaluation CT scan.

## Data Availability

The data used to support the findings of this study are available from the corresponding author upon reasonable request and with permission of the Erasmus Medical Center Rotterdam.
